# Supplementary material for: Acceptability of daily pre-exposure prophylaxis among adolescent men who have sex with men, travestis and transgender women in Brazil: A qualitative study
Source: PLoS One. 2021 May 4;16(5):e0249293. doi: 10.1371/journal.pone.0249293 (PMC8096080; doi:10.1371/journal.pone.0249293)
Supplement: S1 File — São Paulo, Belo Horizonte, Salvador. Brazil, 2019. (DOCX) [file pone.0249293.s001.docx]

**S1 File.** Interview guide for adolescent key informants who participated in the formative research of PrEP 1519 Study. São Paulo, Belo Horizonte, Salvador. Brazil, 2019.

| Component | Prompt | Specific instructions |
| --- | --- | --- |
| Warmer | Could you tell me a bit about your daily life with your family and friends, at school or at work?  Could you tell me a bit about your experiences at work, at school, with your friends and family, etc.? | Initial question to get the interview started (conversation starter - narrative). Ask your interlocutor to talk a little bit about their experiences at work, at school, with their friends. Let them begin or tell their story freely, starting at any point or time in their lives (as they see fit).  Ask them to describe themselves freely.  Let them go into whatever aspects they feel are important. |
| Mapping out social spaces | Could you describe the spaces in this city where you meet up with friends/colleagues/acquaintances who are gay and/or *travesti* adolescents and/or adolescent transgender women?  Could you also describe the spaces in this city where you find sexual partners?  Could you describe the online spaces (internet, mobile apps, etc.) you all use regularly? What is the purpose of using these virtual spaces? Do you use them to find sexual partners? | Explore where the adolescents are and where/in what situations they engage in emotional/sexual interactions;  Ask about who they tend to go out with (alone, in a group, etc.), where they go out to have fun, check out the talent, etc., and any specific places for hooking up;  Find out which social media, which devices (PCs, smart phones, tablets), and which dating/hookup apps they use. |
| Gender identity/expression, sexual orientation, and sexuality | Now, let’s talk about your sexual orientation – who you have sex with or feel attracted to.  How do you usually identify yourself to your friends (gay, queer, transgender, other)?  Do you feel more attracted to men/boys or women/girls?  Do other people know about your sexual orientation and/or gender identity? Who knows? If they don’t know, why haven’t you told them? | Find out about identity categories.  Remember that we have a great diversity of categories (gay, homosexual, bisexual, heterosexual, MSM [a man who has sex with men], queer, queen, cub, bear, woman, gay, etc.), but lots of people don’t identify with any of them.  Remember that gender identity is not necessarily a determinant of sexual desire. |
| Vulnerability to HIV, violence, and discrimination | How do you protect yourself from HIV and STIs in your sexual relations?  Do you normally take drugs or drink when you’re looking for a hook-up or hooking up with someone? If so, does this affect the way you protect yourself against HIV?  Do you think you might get AIDS? Why (not)?  Have you ever been in a dodgy situation, where there were threats or when an argument got out of hand, in any of your relationships or with a hookup?  Do you feel discriminated against in your daily life? Why (not)? For the color of your skin? For your sexual orientation? For your gender identity/expression? For your social class? Do you think this interferes at all in how you interact with partners and/or protect yourself against HIV? | Find out about choices and uses of prevention methods;  Find out about any (potential) experience of violence in emotional/sexual relationships. |
| Knowledge of and motivation to use PrEP and HIV self-testing | Before this study, had you ever heard of the daily use of pre-exposure prophylaxis (PrEP) to prevent getting infected with the AIDS virus? If so, what have you heard about it?  *EXPLAIN WHAT PrEP IS:* Pre-Exposure Prophylaxis is an HIV prevention strategy. A person who doesn’t have HIV takes antiretroviral drugs (the same pills people take to treat HIV) because they have unprotected sex (like sex without a condom). If the pills are taken every day, it will prevent HIV infection. If the person does not take it every day, they may get infected with the virus.  If you wanted more information about PrEP, where or who would you get it from?  Based on what you now know about PrEP, would you be interested in taking this pill every day to prevent getting infected with HIV?  If so, why?  If not, why not?  If you used PrEP, would you feel less afraid of getting infected with HIV?  The side effects are light and only last for a week or so. They include feeling sick, belly aches, vomiting, dizziness, and headaches. Do any of these side effects worry you?  Some people think that people who take PrEP might stop using condoms and have more partners because they think they’re no longer at risk of getting HIV. What do you think about that?  If we decided to advertise PrEP and HIV/STI testing, how and where do you think we should do it? What kind of message do you think we should use to get through to younger people?  Would you be afraid of taking PrEP because other people might assume you had HIV? Why (not)?  Would you be afraid of taking PrEP because other people might think you sleep around? Why (not)?  Have you ever heard of HIV self-testing? (Explain the difference between the finger prick test and the oral fluid test.)  Do you think self-testing might help you and/or your partners to protect yourselves against HIV?  What would be the best way of getting the self-test kit? | Find out about perceptions, knowledge, and motivations related to PrEP.  Find out about knowledge and perceptions of self-testing. |
| Acceptability of the study design and planned recruitment strategies | We’re going to do a study in this city that will offer PrEP to adolescents aged 15 to 19 at at substantial risk of HIV infection. We’re going to monitor them every three months for three years to make sure the PrEP is working. What do you think about that? Would you take part? Why (not)?  I’d like to hear your opinion about how we could work with the following recruitment strategies for our study:   - **Peer interventions**: adolescents trained in prevention methods working together with groups of adolescents and in places where they hang out together; - **Enrollment through the supply of information, prevention material, and testing at social spaces**: weekly interventions by health workers and trained laypersons at public spaces, offering 15- to 19-year-old adolescents rapid testing to diagnose HIV infection, screening for syphilis and hepatitis B and C, prevention materials, like condoms and lubricants, and referrals to health services for HIV and STI prevention, diagnosis and treatment; - **Counseling and testing at an NGO**: health workers and trained laypersons, who will offer counseling and rapid testing for HIV, syphilis, and hepatitis at an NGO (or at another institution, to be selected locally); - **Social media interventions**: interventions made directly by the project targeting the study population, via the website, Facebook page, WhatsApp, and other social media and apps. - **Enrollment via the health service, schools, and PrEP users:** health workers or trained laypersons who offer educational and informative activities geared directly towards the adolescents; health services, schools, and other government and non-governmental institutions will be included in this referral/reverse referral network.   Is there any other strategy you’d like to suggest? |  |
