# Supplementary material for: Acceptability of daily pre-exposure prophylaxis among adolescent men who have sex with men, travestis and transgender women in Brazil: A qualitative study
Source: PLoS One. 2021 May 4;16(5):e0249293. doi: 10.1371/journal.pone.0249293 (PMC8096080; doi:10.1371/journal.pone.0249293)
Supplement: S2 File — São Paulo, Belo Horizonte, Salvador. Brazil, 2019. (DOCX) [file pone.0249293.s002.docx]

**S2 File.** Focus group guide for adolescent key informants who participated in the formative research of PrEP 1519 Study. São Paulo, Belo Horizonte, Salvador. Brazil, 2019.

| **IDENTIFICATION DATA** |
| --- |
| **Date: ____/____/____**  **City: ___________________________________________________________**  **Address of group venue: ____________________________________________**  **Type of venue (room, NGO, clinic, etc.): ____________________________________**  **Group number: _____________________________________________________**  **Brief description of group (who they are, ages, educational level):_________ ____________________________________________________________________________________________________________________________________________________________________________________________________________________________________________________________________________________________________________________________________________________________________________________________________________________________________**  **Start time: _ _._ _ End time: _ _._ _**  **Moderator: ___________________Observer (note-taker): ____________________**  **Layout of group members:**  **[Label each position with a number or letter for each group member, including the position of the moderator and observer. Use this drawing to identify the participants’ speech].**  **Observations:**  **______________________________________________________________________**  **______________________________________________________________________**  **______________________________________________________________________**  **______________________________________________________________________**  **______________________________________________________________________**  **______________________________________________________________________** |
|  |
| **Instructions for coordinator:**  **Before starting the focus group:**   - Clearly describe the goals and stages of the focus group, namely: the informed consent form will be read out (whose acceptance is a prerequisite for joining the group); authorization for recording of the group session will be requested; and a socio-demographic questionnaire will be administered; - Instruct everyone to keep information disclosed within the focus group confidential; - Test the recording: click on “Rec/Pause” to begin a test recording; - After answering any questions, ask the participants if they agree to take part and have their speech recorded. Sign and date the informed consent form, attesting that verbal consent was given by each respective participant; - Ask the participants of the focus group how they want to be identified during the discussion (if they do not want their own names to be used) and ask them to wear labels showing this ID; - The observer (note-taker) must note down the position of each of the members and their respective IDs on the focus group identification form.   **During the focus group:**   - Record the date, time, and venue (city); - Introduce the moderator and observer (note-taker) to the participants and explain their respective roles; - Run the group according to the script, but rather than sticking to a question-answer format, encourage the participants to discuss the questions amongst themselves; - Speak clearly and not too fast – that will make it more likely for the participants to do the same; - Ask the participants to speak up if they start to mumble; - Don’t make any noise with papers, cups, or other objects near the microphone; - Monitor the recorder (discreetly) to make sure it is recording; - At the end, say “Focus group concluded at _____ [time].”   **After the focus group:**   - Thank everyone again for their participation and refund their expenses; - Label all the research material with the same identification code: the digital files, the socio-demographic questionnaires, the informed consent form, and the notes made on the focus group. Use the following code: - Focus group (GF); - Location: BH (Belo Horizonte), SSA (Salvador), and SP (São Paulo); - For the population: HSH (men who have sex with men), Tr (*travesti*), and TGW (transgender women); - Label all printed matter and all audio files with the same identification code (e.g., GFSSAHSH01); - Don’t forget to save the audio file on your computer and upload it to the cloud. |
|  |

- **Introduction of group members**
- **Warmer**
- **To inform: Everyone should be instructed to keep information disclosed within the focus group confidential.**
- **Introduce the project: We’re going to do a study in this city that will offer PrEP to adolescents aged 15 to 19 at substantial risk of HIV infection.**

**EXPLAIN WHAT PrEP IS*:* Pre-Exposure Prophylaxis is an HIV prevention strategy. A person who doesn’t have HIV takes antiretroviral drugs (the same pills people take to treat HIV) because they have unprotected sex (like sex without a condom). If the pills are taken every day, it will prevent HIV infection. If the person does not take it every day, they may get infected with the virus.**

**We’re going to monitor them every three months for three years to make sure the PrEP is working.**

**[Ask the focus group members:] What do you think about a study like that? Would you take part? Why (not)?**

**What would make people your age participate or not participate? Why?**

- **Motivation to use PrEP**

**Would you be interested in taking this pill every day to prevent HIV infection?**

**Could you describe a situation in which PrEP would help you in your relationships?**

**If you used PrEP, would you feel less afraid of getting infected with HIV?**

**The side effects are light and only last for a week or so. They include feeling sick, belly aches, vomiting, dizziness, and headaches. Do any of these side effects worry you?**

**Would you be afraid of taking PrEP because other people might assume you had HIV?**

**Would you be afraid of taking PrEP because other people might think you sleep around?**

**Some people think that individuals who take PrEP might stop using condoms and have more partners because they think they’re no longer at risk of getting HIV. What do you think about that?**

**Acceptability of the study design and planned recruitment strategies**

**[Ask the focus group members to discuss:] We’ve thought of five different ways of getting in touch with adolescent MSM, *travestis* and transgender women to offer them HIV testing and PrEP. I’d like to hear your opinion about them. I’m going to explain them one by one, and then we can discuss the pros and cons of each one.**

- **Peer interventions**: adolescents trained in prevention methods working together with groups of adolescents and in places where they hang out together;
- **Enrollment through the supply of information, prevention material, and testing at social spaces:** weekly interventions by health workers and trained laypersons at public spaces, offering 15- to 19-year-old adolescents rapid testing to diagnose HIV infection, screening for syphilis and hepatitis B and C, prevention materials, like condoms and lubricants, and referrals to health services for HIV and STI prevention, diagnosis and treatment;
- **Counseling and testing at an NGO**: health workers and trained laypersons, who will offer counseling and rapid testing for HIV, syphilis, and hepatitis at an NGO (or at another institution, to be selected locally);
- **Social media interventions**: interventions made directly by the project targeting the study population, via the website, Facebook page, WhatsApp, and other social media and apps.
- **Enrollment via the health service, schools, and PrEP users**: health workers or trained laypersons who offer educational and informative activities geared directly towards the adolescents; health services, schools, and other government and non-governmental institutions will be included in this referral/reverse referral network.

**What kind of message should the project be conveying to young people about PrEP?**

**Find out about:**

**Best times, places, and activities; allowance/refund of the expenses;**

**Privacy and confidentiality for testing and counseling.**
