# Supplementary material for: Acceptability of daily pre-exposure prophylaxis among adolescent men who have sex with men, travestis and transgender women in Brazil: A qualitative study
Source: PLoS One. 2021 May 4;16(5):e0249293. doi: 10.1371/journal.pone.0249293 (PMC8096080; doi:10.1371/journal.pone.0249293)
Supplement: Attachment — Submitted filename: Reviewer Comments.docx [file pone.0249293.s015.docx]

I appreciate the possibility to evaluate the manuscript Acceptability of daily Pre-Exposure Prophylaxis for adolescent men who have sex with men, travestis and transgender women in Brazil: a qualitative study, that has as objective to analyze the acceptability of PrEP among adolescent MSM, travestis and TGW from three Brazilian capital cities.

I find the topic very relevant and quite important to have a perspactive from Latin America and from a country like Brazil where the provision is universal. Overall it si s a very interesting manuscript, providing information on young people, very useful to design future programs that aim at scaling up PrEP.

I will suggest the authors to review the manuscript and its English grammar as, particularly in the results sections, sometimes is a bit difficult to follow. Sentences can be polished and shorten. I provide some examples and it needs to be edited as a whole.

Title

Is appropriate and clear, I think it will read better if it has “among adolescent…” rather than “for adolescent”.

Abstract

It’s appropriate and clear.

Introduction

Is well organised and all relevant information is provided. I appreciate that the authors have explained that in Brazil, as in many South-American countries, the word travesti is linked to a political identity and part of the LGBTi community vindicate this identity.

I would suggest to add some lines on the public policies of Brazil in terms of ARV and PrEP provision. In other countries, not having universal access and gratuity can be considered to be the main barrier, however, this manuscript shows that there are many other issues and misconceptions to work with young people and sexual minority groups.

In line 63, authors mentioned “The literature describes different socio-structural, personal and programmatic barriers to adolescents’ access to HIV services…”, it seems that authors describe 3 types of barriers, but according to my understanding and the description provided, programatic barriers should be included in structural barriers… in line 70, interpersonal barriers, that were not mentioned in that introductory sentences, are described… perhaps this can be polished and talk about personal, social and structural barriers… or another categorisation but provide the description using same category.

Line 84-85 review the sentence “high acceptability of PrEP has been observed in many investigations, and facilitators to it include free access to PrEP”… “facilitators to it include” may be replaced by “the main facilitators were” or another wording.

Line 89-90 authors mentioned “The meanings that the adolescents attribute to processes relevant to the acceptability of PrEP include…”, however, the description includes a series of aspects, such as sexual orientation or autonomy, that are not meanings of… probably this sentences can be rewrite and simplify… “Relevant aspects/issues/ topics…. For prep acceptability among adolescents are…. “

In line 95, authors mentioned “life contexts and sexual relationships”… as it is currently written, it seems as a separate set of variables… but I understand that authors intended to explore the role of life contexts and sexual relationships on Prep acceptability. I suggest to rephrase this sentence

Methods

Overall is very complete but it is difficult to follow… I would only suggest to add some subtitles to organise the information: participants, materials/instruments, procedure, data analyses, ethical considerations/statement…or the like.

Authors mention 1) mapping, 2) interviews and 3) focus-groups…. Can you describe better how you organised the mapping?

It is not quite clear how participants were recruited . The section starts with “after mapping out spaces of social interaction though participant observation”, which is not vey clear,… and finished with Participants were recruited in public and private high schools, and adolescent LGBT venues. Can you clarify the link or sequence between “mapping spaces”, “participants observation”, “schools and venues”,…

In Table 1, the word “transvestite” rather than travesti is written, It would be better to change it for consistency.

Interviews and focus groups used the same methodology?  the interview guide was the same for both interviews and goops?

Results

The section is quite long and it can be easily shorten by removing narratives that point to the same explanation. There also are repetitive explanations, for example with transactional sex, that can be presented in one paragraph and provide one or two narratives as examples.

- In the description of participants, both in text and in Table 2, bisexual, asexual, etc.. have double “s”. Ej: panssexual, should be pansexual, bissexual, should be bisexual…

Lines 225-228, the explanation is clear but the three examples are quite similar and non of them refer to doubts of drug interaction, for instance. I suggest authors to change some of the reiterative narratives and replace for a diferente example.

Lines 251-252, talk about prejudices and misconceptions related to PreP use that act as barriers to its accessibility, however, the example provided, is a bit long and reiterative and not very representative of the interpretation. This can be polished.

Line 263 “credited PrEP with additional protection against STIs” I will clarify that this is a misconception.

Line 275… review the wording of the following sentence “ especially with casual sex as this raised more concern about STIs” …. “be it because”

Discussion

This section is very interesting, particularly in the discussion of ideas, meditating process and the like. But it seems to be written by a different person, different English, writing style… and also, some of the thinking provided, are not well presented in the results section. Thus, I will suggest to review the coherence between both sections.

I found the first paragraph not at all supported by the testimonies provided but for the feeling and interpretations of researchaers… “feeling and anguish” …“distrust of sexual healthcare providers”. These ideas seems not to be presented in the results section, or, if they are, it seems to me that they were not the main barriers, at least, not presented in that manner. You can revise the wording.

Line 546-47 mentioned “Other studies have also found that young people perceived low levels of self-efficacy in terms of managing their routine to take PrEP on a daily basis” the references for that should be provided precisely in the discussion.

Line 559-561 can be moved to a limitation section

The paragraph on “continuous use of medication”, not only is very long, but it is not well articulated with the rest of the discussion neither with the results section… it also seems to be written with a different English. Thus, I suggest to shorten it and probably make some of this interpretations in the results section.

Similar impression with the next sections of the discussion.

A section with limitations and suggestions for future research should be added (short and simple, no more than one paragraph).
